# Identification of a Topological Characteristic Responsible for the Biological Robustness of Regulatory Networks

**DOI:** 10.1371/journal.pcbi.1000442

**Published:** 2009-07-24

**Authors:** Yangle Wu, Xiaomeng Zhang, Jianglei Yu, Qi Ouyang

**Affiliations:** 1 Center for Theoretical Biology, Academy for Advanced Interdisciplinary Studies, Peking University, Beijing, People's Republic of China; 2 The State Key Laboratory for Artificial Microstructures and Mesoscopic Physics, School of Physics, Peking University, Beijing, People's Republic of China; 3 Department of Physics, Hong Kong Baptist University, Kowloon Tong, Hong Kong, Special Administrative Region, People's Republic of China; Washington University in Saint Louis, United States of America

## Abstract

Attribution of biological robustness to the specific structural properties of a regulatory network is an important yet unsolved problem in systems biology. It is widely believed that the topological characteristics of a biological control network largely determine its dynamic behavior, yet the actual mechanism is still poorly understood. Here, we define a novel structural feature of biological networks, termed ‘regulation entropy’, to quantitatively assess the influence of network topology on the robustness of the systems. Using the cell-cycle control networks of the budding yeast (*Saccharomyces cerevisiae*) and the fission yeast (*Schizosaccharomyces pombe*) as examples, we first demonstrate the correlation of this quantity with the dynamic stability of biological control networks, and then we establish a significant association between this quantity and the structural stability of the networks. And we further substantiate the generality of this approach with a broad spectrum of biological and random networks. We conclude that the regulation entropy is an effective order parameter in evaluating the robustness of biological control networks. Our work suggests a novel connection between the topological feature and the dynamic property of biological regulatory networks.

## Author Summary

Living organisms exert very complicated control on the functionality of their components. Such control systems can often operate in a surprisingly robust manner, in spite of constant perturbations from fluctuating internal conditions and a volatile external environment. What feature makes such control mechanisms robust? Is there a general way to achieve robustness? Here, we address these questions by investigating the wiring of interaction networks, which contains the most condensed information about the control mechanisms of biological systems. We suggest that one of the most important factors in the realization of biological robustness rests in the global coherency of the control strategy, i.e., the consistency of commands flowing through different routes in the network to the same destination. To implement this idea, we propose an order parameter termed ‘regulation entropy’ to quantitatively describe this control consistency of networks. We find that this order parameter correlates with the resistance of the system to external perturbations as well as internal fluctuations. Our results suggest that the self-consistency of the control strategy is important for the vitality and robustness of living organisms.

## Introduction

Biological regulatory networks play an essential role in all living organisms. The investigation of their general behaviors is an important subject in the current research of systems biology. Recently, the reliable functionality of these networks has attracted much attention [1]; it has been widely recognized that some important biological networks are globally stable against external perturbations and can perform their functions without much fine-tuning of their internal parameters [2]–[5]. These properties of biological control systems are well demonstrated by the recent successful works on the Boolean approximation of regulatory networks [6]–[8]. It was shown that a bare Boolean dynamics is often good enough to describe the essence of biology. Moreover, biological networks simplified by the Boolean approximation often still show a significant dynamic stability, characterized by their global attractors of the biological stationary states and the stability of the biological pathways [7]–[9].

It is widely believed that the topological properties of a biological control network largely determine its dynamic behavior. Therefore, the robustness of biological systems should have its root in the special arrangement of the links in the control networks. Several authors have made various attempts to quantitatively identify this structural origin of network robustness, yet their studies were mainly focused on the distribution of connections, such as the scale-free distribution of degrees [10], or the modularity [5], while the function of links was totally overlooked. Although these studies provide important insights into the emergence of biological robustness, their descriptions miss the important ingredient of the network systems and thus are incomplete. In this paper, we try to make a step forward in this line of researches by including the sign (positive/negative) of links. To this end we define a new order parameter, named regulation entropy, to measure the signed topology of a biological network. Using the cell-cycle control networks of the budding yeast (*Saccharomyces cerevisiae*) and the fission yeast (*Schizosaccharomyces pombe*) as examples, we first show that this parameter puts a constraint on the robustness of the two control networks, then we provide additional evidences showing that it can serve as a good indicator of the robustness of biological control systems in general.

## Results

### Definition of the Regulation Entropy

It is well known that the components of a biological system are often connected by complicated interactions, such as binding, (de)phosphorylation, transcription, synthesis and degradation. To model such intricate systems, one needs to employ various approximations. Guided by the balance between model accuracy and computational efficiency, one may neglect the details of biochemical kinetics while preserve the crucial regulatory relations among key players of the original interaction network. Specifically, lots of biochemical interactions are realized by a cascade of reactions, with the fate of the final product almost completely determined by the upstream signal. In practice, such an indirect interaction between the upstream and the downstream of a cascade is often simplified into a direct link in network modeling, especially for the Boolean case. Thus it does not make much sense to take too seriously the difference between direct and indirect interactions on an interaction map. From this point of view, we should place direct links and indirect ones in a biological network on a more or less equal footing. On the other hand, the regulation coherency of the control network is often an essential property of the system. Here coherency denotes the situation that commands from different controllers do not contradict, but to strengthen each other. Based on these considerations, we introduce an order parameter to describe the structural or topological property of a biological control network.

In a simple form, a biological regulatory network can be expressed in a signed directed graph. The nodes of this graph represent biomolecules in the system, and the directed edges denote interactions between these biomolecules, with positive and negative signs indicating up and down regulations respectively. For example, a link 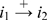 may represent the fact that the transcription factor 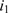 promotes the synthesis of the protein 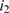, or the protein 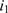 activates the protein 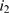, etc. Following the direction of arrows, one may take a ‘walk’ on the graph. And a *path* can be conventionally defined as a self-avoiding walk on the graph, i.e., *distinct* nodes sequentially connected by arrows present in the graph. To handle self-interactions, this definition can be extended to allow the starting and the ending nodes to be the same, while still require intermediate nodes to be distinct from each other and from the starting node. Each path from node 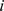 to node 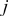 represents a regulation pathway from node 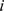 to node 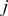. The overall regulation effect of a path may be either positive (up) or negative (down), depending on the sign of each link and the total number of links in the path. For example, a chain composed of an even number of negative regulations behaves like a positive regulation. We thus can associate each path with a sign, which is determined by the product of the signs of all the links in the path. By this definition, a path becomes a concrete representation of regulation in general, both direct and indirect.

**Figure 1:**
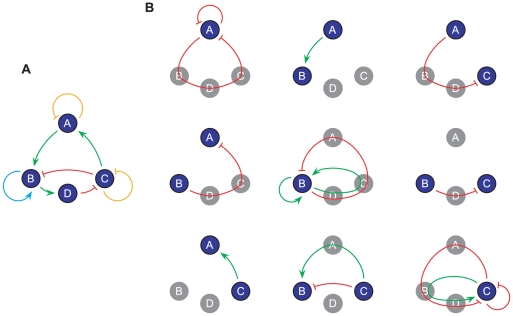
An illustrative example of the calculation of the regulation entropy. The network is shown in A, and the sets 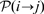 are listed in B. Note that the node ‘D’ is trivially connected, and thus is ignored in the calculation from the very beginning. Green arrows and red blunt-end ones are activating and inhibiting interactions, respectively. For self-pointed arrows in A, orange blunt-end indicates self-degradation, whereas cyan indicates self-activation.

Next step we define 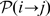 as the set of all paths from node 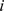 to node 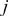. Obviously, elements in the set 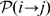 may carry different signs, which means that the regulations of node 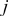 by node 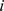 may be self-contradictory, with some components activating and others inhibiting node 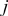. In this case, the overall effect of regulation would delicately depend on the coupling of all these components, and thus more sensitive to the details of interactions and the status of intermediate nodes, especially when they are on the overlap with other sub-circuits. However, if most of the paths in 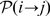 have the same sign, there would be less potential conflicts among the instructions sent from node 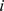 through different routes to node 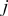. As a result, we can expect a reliable regulation that is insensitive to biochemical details and leads to a relatively ordered dynamic behavior of the network. To quantify this ambiguity of interactions, we define the regulation entropy 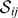 for each pair of node 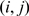 that is connected by at least one path from node 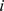 to node 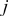:
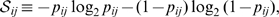
in which 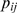 is the ratio of positive paths in 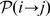. If 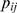 tends to zero or one, 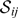 will tend to zero, which is the minimum of this function; whereas 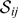 reaches its maximum value of 1 at 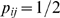, which corresponds to the cases of most probable conflicts among the instructions sent from node 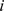 through different routes to node 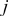. It is natural to introduce the entropy 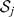 of node 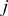 by averaging 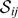 over regulators of node 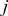. Then, we can use the averaged entropy 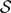 of the nodes in a network as a measure of the entropy of the whole network; we name it the regulation entropy of the network. We should point out that the nodes that have only one incoming link and one outgoing link should be excluded during these averaging processes. In this way, the value of the regulation entropy will be invariant under trivial transformations like inserting a node into the middle of an existing link, i.e. changing 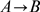 into 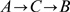, and vice versa. Figure 1 provides a concrete example. Evidently, from the definition we have:
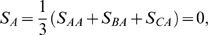


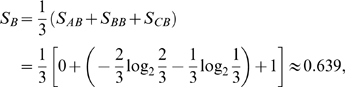


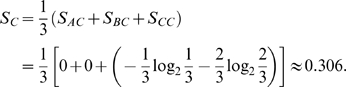



Thus the regulation entropy of the network in Fig. 1**A** is:
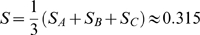



Admittedly, different regulations may have diverse timescales, which makes the coupling of different chains of interactions non-trivial, as shown in the analysis of the function of various feed-forward loops [11]. But to construct an order parameter describing the overall coupling coherency of networks, it is justifiable to take a coarse-graining approach, assuming that the difference of interaction details can be neglected in a first approximation. In the following, we will demonstrate the relevance of this crudely constructed quantity 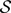 to the functional and dynamic properties of some real biological control systems.

### The Regulation Entropy Characteristic of Biological Networks

**Figure 2:**
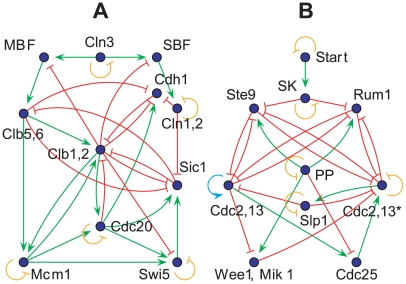
The simplified cell-cycle networks. The cell-cycle control networks of the budding yeast [7] and the fission yeast [8] are shown in A and B, respectively. Green arrows and red blunt-end ones are activating and inhibiting interactions, respectively. For self-pointed arrows, orange blunt-end indicates self-degradation, whereas cyan indicates self-activation.

We use two specific examples, the cell-cycle regulatory networks of the budding yeast and the fission yeast, to investigate the relation between the regulation entropy and the functional and dynamic properties of biological networks. As pointed out by Davidich *et al* [8], these two simplified cell-cycle control networks (see Fig. 2) are diverse in nature, providing an ideal test-bench for the investigation of general properties of network dynamics. Extensive literature has been devoted to the construction of Ordinary Differential Equation (ODE) models [12],[13] as well as Boolean network models [7],[8] to reveal the dynamic properties of the underlying control systems. To avoid getting lost in the details of the parameter-setting of the ODE models, we use the Boolean approach to gain a first impression of the effect of the regulation entropy on network dynamics. For a brief introduction to Boolean network dynamics and a recapitulation of the two model networks, see *Materials and Methods*.

The behavior of these two biological networks is compared with randomly generated networks. Following Lau *et al* [9], we refer to the combined structural and functional ensemble for this comparison, i.e., the ensemble of networks that have the same number of connections as the corresponding cell-cycle network and can produce the same Boolean sequence of the corresponding cell cycle. For succinct reference, we shall denote this kind of ensemble by 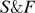 in subscript. Specifically, we employ the basic procedure described in Ref. [9] to generate 10^6^ samples from each of the 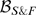 and 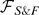 ensembles of candidate networks, where 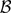 and 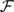 denote the budding yeast and the fission yeast, respectively.

**Figure 3:**
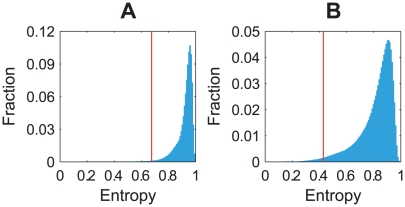
Distribution of the regulation entropy in the cell-cycle random ensembles. Histograms show the regulation entropy distributions of the combined structural and functional ensembles of A the budding yeast and B the fission yeast. 10^6^ networks were sampled from each ensemble. The vertical line denotes the position of the corresponding cell-cycle network.

First, we check the regulation entropy distribution of the random networks and the position of the corresponding cell-cycle network in the distribution. Figure 3 summarizes the results. One observes that most random networks have high regulation entropy values, while those of the two biological control networks are ranked among the lowest 1% or so. Considering that these two biological networks are fundamentally different in the control mechanism (strongly damped vs. auto-excited, transcriptional vs. translational) [8] and are diverse in their average connectivity and their ratio of negative links, the departure of the biological networks from the majority of random networks may be quite general. And the regulation entropy may reveal an important topological characteristic of biological control networks in general.

All of the networks in the 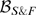 and 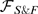 ensembles can produce the right cell-cycle trajectory. However, they are diverse in their ways to fulfill the function. The high regulation entropy values indicate that most of these random networks are sending self-contradictory commands, and it is probably the crude Boolean approximation that covers up these inconsistencies by totally suppressing interactions from the nodes that are not ‘active’ enough, and therefore produces the trajectory as it happens. In contrast, biological networks have delicate wiring, with most of their components well tuned, as suggested by its distinctively low regulation entropy. This makes it more likely that a subset of interactions can represent the overall effect of regulation. This redundancy enables the cell-cycle networks to reliably produce the target trajectory.

### Correlation with Dynamic Stability

In this and next sections, we discuss in detail the correlation of the regulation entropy with the robustness of the yeast networks. From the point of view of nonlinear dynamics, the robustness of a system means that it is stable against external perturbations on the state of the system (state or dynamic stability), and it is stable against perturbations on its control parameters (structural stability). Here, we measure the state stability by the basin size of the biggest attractor of the dynamic system [7] and the network sensitivity [14] in the Boolean model, and we measure the structural stability by the Q value of parameter insensitivity in the ODE model [5].

Previous studies on the Boolean models of the yeast cell-cycle networks showed that these systems are globally stable in dynamics [7]–[9]. Observing that such networks are characterized by their low regulation entropy, we investigate the relation between the regulation entropy and the dynamic stability of these networks. To this end, we calculate the regulation entropy, as well as the basin size of the biggest attractor (we shall call it ‘basin size’ for abbreviation) and the network sensitivity [14] for the networks generated from the 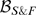 and 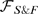 ensembles. (For a brief review of the definition and implication of these dynamic properties, see *Materials and Methods*.)

**Figure 4:**
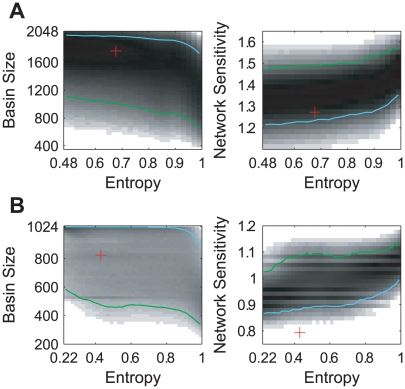
Correlation of the regulation entropy with dynamic stability in the cell-cycle random ensembles. The density profiles show the correlation between the regulation entropy and the dynamic properties of the combined structural and functional ensembles of A the budding yeast and B the fission yeast. Linear gray-scale with respect to the logarithm of density is adopted to enhance visibility. The red cross denotes the position of the corresponding cell-cycle network. The green and cyan outlines show how the levels of the least robust 5% and the most robust 5% vary with the regulation entropy.

To compensate for the highly unbalanced distribution of the regulation entropy and to get a well-rounded estimation of the dependence of the dynamic properties on 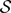, we divided the [0,1] interval of the regulation entropy into equal segments of length 0.02, and randomly sampled 10^5^ networks from each of the segments. The correlation of the dynamic stability with the regulation entropy is shown in Fig. 4. The green and cyan lines indicate the bottom 5% and the top 5% levels of robustness, respectively. These skew outlines show that networks with relatively low regulation entropy tend to have relatively stable dynamics, i.e. the lower regulation entropy is, the larger basin size and the lower network sensitivity they are most likely to have. This positive correlation is evidently expectable, since redundancy enhances robustness.

### Correlation with Structural Stability

The parameter insensitivity or structural stability of a network is an important facet of the robustness of the system. The discussions in the previous section are based on the synchronous Boolean approximation of chemical kinetics, which already implies the parameter insensitivity of the systems. To discuss the structural stability of the two cell-cycle networks, we need to use continuous models based on Ordinary Differential Equations (ODE). Previous studies have shown that some biological networks are extremely insensitive to the variation of parameters. For example, it has been demonstrated that a large proportion of parameter space can support the proper functioning of the *Drosophila* segment polarity network [2]–[5].

Here, we should point out that dynamic stability and structural stability characterize different properties of a network system, although they may in some cases be correlated, as pointed out by Ciliberti *et al* [15]. In general, dynamic stability addresses the resistance of a biological state or a biological pathway to external perturbations, while structural stability measures the functional stability of a system under internal fluctuations of parameters.

In this section, we discuss the relation between the regulation entropy and the structural stability of biological networks. For this purpose, we carried out extensive simulation of the ODE models of the two cell-cycle systems, and compared the results with the behavior of random networks. For each network, we randomly selected a set of control parameters, and checked if the system can perform its biological function (following the biological pathway). By repeating this process we got an estimation of the 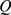 value of the network, which is defined as the fraction of the parameter space that can perform the biological function [2],[4],[5]. For details regarding the simulation of the ODE models and the functionality judgment, see *Materials and Methods*.

Since only an extremely tiny fraction in the huge network configuration space can fulfill the cell-cycle function [9], we limited our simulations to the networks that can produce the cell-cycle sequence in the Boolean scheme. Moreover, in order to rule out networks with an unrealistically large number of connections, we fixed our scope to the networks with the same number of connections as the corresponding cell-cycle network, i.e., we focused on networks in the 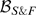 and 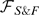 ensembles.

**Figure 5:**
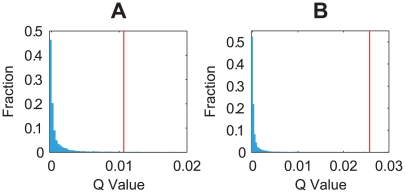
Distribution of the 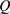 values in the cell-cycle random ensembles. The histograms show the 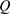 value distribution of the combined structural and functional ensembles of A the budding yeast, and B the fission yeast. 10^4^ networks were sampled from each ensemble, and 10^4^ parameter sets were sampled for the dynamics simulation of each network. The vertical line denotes the position of the corresponding cell-cycle network.

Figure 5 gives the 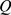 value distribution of these random ensembles, and the position of the corresponding biological network in the graph. One observes that the two cell-cycle networks have very high 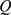 values (about top 1%), even among the networks that can support the cell-cycle function under the Boolean approximation. This provides further examples of parameter-insensitive biological networks.

**Figure 6:**
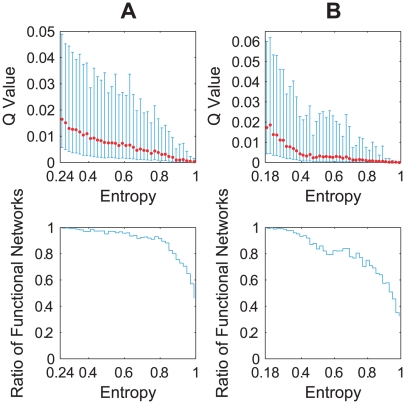
Correlation of the regulation entropy with parameter insensitivity in the cell-cycle random ensembles. The two error bar plots show the dependence of the 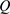 value on the regulation entropy, with the lower and the upper error bars showing the standard deviation of the lower and the upper halves from the average value (red dot), and the two stair plots indicate the dependence of the ratio of functional networks on the regulation entropy, in the combined structural and functional ensemble of A the budding yeast and B the fission yeast, respectively.

More importantly, our calculation shows a strong negative correlation between the regulation entropy and the 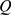 value (and thus structural stability), which is more evident if we check the ratio of ‘functional networks’, i.e. the fraction of networks with at least one parameter set that can pass the functionality judgment in the simulation [5]. In this calculation, we divided the [0,1] interval of the regulation entropy into equal segments of length 0.02, and randomly sampled 500 networks from each of the segments to estimate the 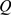 value and the ratio of functional networks, with 10^4^ parameters tested for each network. Figure 6 shows the calculation results. We believe that this correlation originates from the essence of the regulation entropy as a measure of conflict among individual interactions: networks with lower entropy, i.e. more consistent coupling of interactions, would have less dependence on the details of the relative strengths of interactions, and thus enjoy a larger degree of freedom in their parameters. It is generally accepted that the structure of a network defines its dynamics; the regulation entropy we propose captures one of possibly many conditions on network structure under which the dynamic stability and the structural stability arise.

### Beyond the Cell-Cycle Systems

Up till now, we have exemplified our theory with the two cell-cycle control networks. The fundamental difference in their control architectures makes it reasonable to expect that the above results may generally hold for other circuits of biological control systems that demand high functional reliability. For this purpose, we used additional four well-studied biological networks to test our theory. The networks include the guard cell abscisic acid signaling network in plants (ABA) [16],[17], the T cell receptor signaling network (TCR) [18], the survival signaling network in T cell large granular lymphocyte leukemia (T-LGL) [17],[19], and the network of physical interactions between nuclear proteins in the budding yeast (PI) [20],[21]. For each of the networks, we calculated the value of the regulation entropy of the system and checked its relative rank in the corresponding background distributions. (For more details regarding these additional networks, see *Materials and Methods* and the supplementary online material Tables S1, S2, S3, S4, S5, S6.)

As these networks have highly non-trivial functions, we did not introduce any functional constraint in the random ensembles. Instead, for each biological network, we generated more than 10^5^ random networks with the same number of activation and inhibition link as in the real network, and we kept constant the in- and out-degree of each node as well. Table 1 presents the calculation results of the relative rank of the regulation entropy values in the corresponding background ensembles for each biological network. For comparison, we also list the results of the two cell-cycle networks (abbreviated as 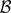 and 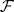). One can see that these diverse biological systems, ranging from signal transduction pathways to the physical interaction network of proteins, also exhibit relatively low values of the regulation entropy. This provides further evidences that biological control networks in general possess relatively low regulation entropy. (For more details about the randomization algorithm, see *Materials and Methods*.)

**Table 1:** The regulation entropy characteristics of some biological systems.

Network	# of Nodes	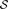	Rank
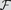	10	0.429	5.2×10^−3^
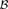	11	0.676	3.1×10^−2^
ABA	39	0.384	7.8×10^−2^
T-LGL	51	0.867	3.4×10^−2^
PI	80	0.528	8×10^−6^
TCR	94	0.539	9.2×10^−2^

**Figure 7:**
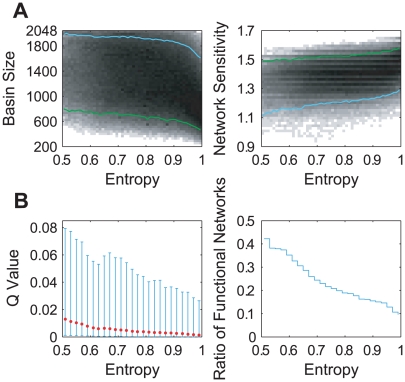
Correlation of the regulation entropy with robustness in the random-function ensembles. The density profiles in A show the correlation between the regulation entropy and the dynamic stability. Linear gray-scale with respect to the logarithm of density is adopted to enhance visibility. The green and cyan lines show how the levels of the least robust 5% and the most robust 5% vary with the regulation entropy. In B, the error bar plot shows the dependence of the 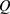 value on the regulation entropy, with the lower and upper error bars showing the standard deviation of the lower and upper halves from the average value (red dot), and the stair plot indicates the dependence of the ratio of functional networks on the regulation entropy.

The next step is to check whether the observed correlation between the regulation entropy and robustness also holds generally. We could not study this correlation in any of the above large-scale networks, since all of them are too huge for the calculation of the global dynamics. Instead, we randomly generated 100 trajectories in the phase space of a 11-node network, each of them having 11 steps ending with a fixed point. We then built the combined structural and functional ensembles derived from each of them as we did with the budding yeast cell cycle, and calculated their distributions of the basin size, the network sensitivity, the Q value and the ratio of functional network. Figure 7 summarizes the calculation results. It shows that networks with relatively low regulation entropy tend to have relatively stable dynamics and low parameter sensitivity, as in the cell-cycle control networks.

## Discussion

In this work, we defined a novel order parameter, the regulation entropy, to characterize the signed topology of regulatory networks, and showed that in general biological networks have very low values of 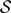. We also established a link between network topology and robustness via this order parameter. First, we identified the correlation between the regulation entropy and the dynamic stability of networks; i.e., a coherent regulation structure of a network will lead to a relatively stable dynamic behavior. Second, we showed an association between the regulation entropy and the parameter insensitivity, which is another aspect of biological robustness concerning the resistance against structural perturbations, i.e. the structural stability.

In the perspective of system biology, these results can shed new light on two important but pending questions. First, why can the yeast cell-cycle control networks be successfully modeled by Boolean networks [7],[8]? Our study suggests it is the extremely low regulation entropy that guarantees large arbitrariness in the choice of parameter, and thus makes the Boolean approximation successful. Second, how do the yeast cell-cycle control networks achieve convergent dynamics and guarantee a globally attracting stationary state? Our work indicates that these networks achieve dynamic stability partly by arranging the coupling of components to guarantee low regulation entropy and thus relatively convergent dynamics. Actually, Lau *et al* [9] already pointed out that the functional constraint of the budding yeast cell-cycle spurs networks to have larger attractor basin, which partly shows the origin of the large basin size of the cell-cycle regulatory network. Our results further illuminate this scenario by identifying the regulation entropy as another source of the attractor enhancement.

Several remarks are in order. First, our results emphasize the significance of the coherent coupling of interactions, while Mangan *et al* pointed out that special functions realized by incoherent feed-forward loop, such as non-monotonic input [22] and the acceleration of response time [23], are common in biological control. These apparently conflicting observations, however, are actually complementary, because they address different facets of the intricate relationship between structure and function. For circuits carrying out specific subsidiary functions, delicate designs such as incoherent feed-forward loops prove to be convenient and powerful, realizing special functions with relatively simple construction. But these subtleties may depend more on the fine-tuning of interaction details, and more likely to fail if intermediate nodes are subject to external control when embedded into a larger system. Such strategy of achieving function at a cost of robustness may be well suited for certain purposes, but might be improper for core networks that have to operate with great reliability and stability, against strong internal as well as external noises. In the latter scenario, networks with low regulation entropy would probably rule.

Second, previous studies on the relationship between structure and function mainly focused on the dynamic effects of feedback loops, from early work of Thomas *et al* [24] to more recent articles of Sontag *et al* [25] and Kwon *et al* [26], providing mathematical explanation and detailed estimation of the phase space structure of the Boolean dynamics. Our work, however, is aimed at elucidating the emergence of general robustness observed in biological networks. The correlations identified within and beyond Boolean models justify our approach of comprehensively checking the consistency of indirect regulations, rather than limiting our scope to feedback loops.

**Figure 8:**
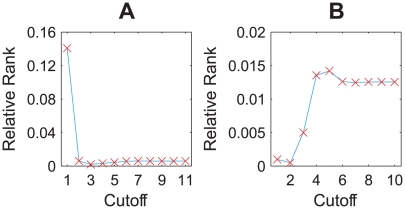
Cutoff insensitivity of the regulation entropy. The red crosses show that the relative rank of the regulation entropy of the biological network in the combined structural and functional ensemble of A the budding yeast and B the fission yeast converges rapidly as the path-length cutoff increases.

Finally, we address a technical issue concerning computational feasibility. One may note that the calculation of regulation entropy might be handicapped by computational complexity. It requires the exhaustive enumeration of paths on a directed graph, which seems to limit its application to large-scale networks. However, if we are only interested in the relative rank of a network in an ensemble, we can introduce a cutoff on the length of paths that we take into consideration, ignoring contributions from longer paths to the regulation entropy. Our study shows that the relative rank of the regulation entropy of a network is not very sensitive to this cutoff on path length (see Fig. 8).

## Materials and Methods

### Boolean Networks

We adopt the most simplified model similar to those in Refs. [7],[9]. The activity of a node is discretized into a binary bit: 0 denotes inactivity; 1 denotes activity. In this way, the state of the whole system can be cast in a Boolean vector, which is evolved forward by the network in discrete time steps, according to specific updating rules. A straightforward setting for such rule is the synchronous ‘majority vote’ updating: assigning +1/−1 weight to each incoming activation/inhibition link, and updating the state of all nodes at once by turning them on/off according to the sign of the simple sum of the inputs from the node active at the previous time step [7]. This is actually a special case of the threshold network model [27]. A slightly modified version of this rule assumes the dominance of incoming negative regulations over positive ones, since it is widely observed in biological networks that inhibition is often much stronger than activation. But self-degradation should still be overruled by incoming activations, if any. We call this latter model ‘strong inhibition’ for reference.

### The Dynamic Properties of Boolean Networks

All the transitions governed by a network of 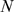 nodes form a flow pattern in the phase space constituted by 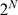 states, with each trajectory ended in an attractor (either a limit cycle or a fixed point) [7],[25]. The basin size of an attractor is the number of states flowing into it. A large basin size of the biological steady state is an indication of the system's stability against state perturbations [7],[8]. Besides, If the Hamming distance over the phase space is introduced as the number of different digits of two Boolean vectors, the network sensitivity 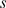 can be defined as the average Hamming distance of the state pairs evolved one step from all of the 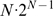 Hamming neighbors, which quantifies the dynamic order of the system: higher 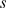 indicates more chaotic dynamic behavior of the system, and 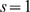 is a critical point separating ordered and chaotic phases [14].

### The Biological Networks

In this work, we performed experiments on the following biological networks. (See the supplementary online material for the detailed documentation of the signed topology of these networks.)

First, we used the budding yeast cell-cycle network model of Li *et al* [7] with 11 nodes, as shown in Fig. 2**A** and Table S1. We adopted the ‘majority vote’ updating rule, in accordance with the original work, which produces a global attracting trajectory resembling the actual sequence of the budding yeast cell cycle. (For more details, see Ref. [7].) We should point out that all the investigations under the ‘strong inhibition’ model give virtually the same results (but not listed here), which shows the independence of our results on the details of the Boolean model.

Second, we used the Boolean model of the fission yeast cell-cycle network with 10 nodes [8], as shown in Fig. 2**B** and . The original work used a similar updating rule as Ref. [7], but introduced non-zero thresholds for the nodes Cdc2/Cdc13^*^ and Cdc2/Cdc13, to guarantee the fulfillment of a trajectory similar to the cell cycle. Here, we adopted an alternative solution, introducing an additional self-degradation for the former, and a self-activation link for the latter. Then, the ‘strong inhibition’ rule produces exactly the same trajectory as Ref. [8]. We made this choice of updating rule merely for convenience in generating random networks, avoiding random shift of thresholds.

In addition, we used another four networks without discussing their dynamics. The first is the guard cell abscisic acid signaling network in plants (ABA). It was first synthesized in the Figure 2 of Ref. [16] from experimental literature. Following Ref. [17], we amputated the nodes without a regulator, but we kept the node ‘ABA’ denoting the upstream signal of abscisic acid in our simplified 39-node network, as shown in Table S3. The second is the T cell receptor signaling network (TCR) shown in Table S4. It was built from the logic model described and validated in Ref. [18] (see its Fig. 2 and Table S2). The third is the survival signaling network in T cell large granular lymphocyte leukemia (T-LGL). It was constructed in Ref. [19] (see its Fig. 1), and simplified in Ref. [17]. We note that in the original network in Ref. [17], the ubiquitous outgoing inhibitions from the conceptual node ‘Apoptosis’ constitutes more than half of the total inhibition links. In order to limit the artifacts that may arise in randomization, we deleted the links starting from ‘Apoptosis’ in our version of this network, as shown in Table S5. We note that TCR and T-LGL only share three nodes, and thus are not redundant but addressing distinct aspects of the T cell biology. The fourth is the 80-node network of physical interactions between nuclear proteins in the budding yeast (PI), shown in Table S6. It was taken from the Fig. 1a of Ref. [21], which is a simplified version of the 329-node network in the Fig. 1 of Ref. [20].

### The Randomization Algorithm

We adopted a systematic reshuffling algorithm for the randomization of signed directed networks. First, two connected pairs of nodes are randomly selected, and then they are randomly rewired by switching the two ending nodes or the two starting nodes with equal probability, as long as no multiple edges form between the same pair of nodes; for example, 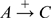 and 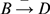 are rewired into either 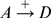 and 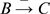, or 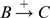 and 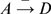. This procedure preserves the total number of inhibitions/activations, and keeps constant the in- and out-degree of each node. The repeated application of this reshuffling, starting from the biological network, enabled us to probe the regulation entropy characteristics of the background ensembles of biological networks, and we set the number of reshuffling steps between two adjacent samplings comparable to the square of the number of nodes, so as to ensure the whole configuration space of the relevant networks is well sampled. Yet we did not use this routine for the cell-cycle networks due to its low efficiency to carry out the functional constraint; instead, we adopted the efficient algorithm developed in Ref. [9] for the cell-cycle networks. And we should point out that for the cell-cycle networks, the ensemble formed by the above random-walk algorithm and the combined structural and functional ensemble constructed in Ref. [9] have almost the same distribution of the regulation entropy.

### The Generation of Random Trajectories

The random *M*-step trajectories in the phase space of an *N*-node network were constructed as follows. For each node in the network, two different moments are randomly selected from the points of time 1, 2, … 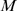, and the time series of the states of this node are set in the following manner: either the states between the two moments are set ‘on’ and the rest moments ‘off’, or the states in between are set ‘off’ and the rest moments ‘on’, with equal probability. Repeating this procedure for each node in the network results in a cascade of activation [9], and we set the ending state of the system to be a fixed point. We note that this construction captures the main characteristics of the Boolean trajectories produced by real biological networks, that the state of each node does not flip frequently (noise-dominated), but to vary orderly and slowly (regulation-dominated).

### The ODE Model

We modeled each of the 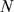 nodes of a regulatory network by an ODE with a self-degrading term characterized by a variable timescale, and each regulation between nodes by an independent Hill function term, with variable strength, threshold and stiffness, and we modeled multiple regulations as the sum of individual regulations. Such translation into ODEs looks crude compared with the delicate cell-cycle models [12],[13], but it provides a systematic way to model the dynamic behavior of random networks.

After non-dimensionalization similar to that in Ref. [5], we arrived at an equation for each node 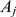,
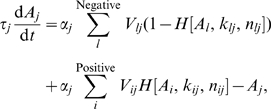
(1)with the Hill function defined as
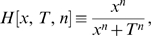
(2)and the normalization constant 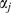 given by
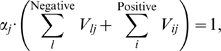
(3)in which we summed over negative and positive regulators for each node 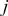. Additionally, we modeled the absence of self-degradation as a positive self-regulation term.

In this set of ODEs, we had 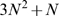 independent parameters: 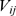, 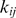, 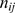, and 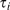. In the random setting of these parameters, we used Latin Hypercube sampling [28] to ensure the minimal correlation between different dimensions of the parameter space. The ranges of the parameters to sample were set as follows: 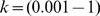, 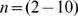, 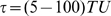 (dimensionless time unit), 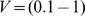, with 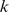 uniformly sampled on the log scale and others on the linear scale, in accordance with previous studies [5]. Then, we employed the function *rkf45* in the GNU Scientific Library [29] to solve these ODEs by numerical integration for a simulation time of 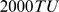, from initial states set according to the Boolean sequence of the cell cycle: specifically, the concentration of initially active nodes is set to 1 and the rest 0.

### Functionality Judgment and the 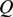 Value Estimation

For a dynamic function (trajectory) in the form of activation cascades like the simplified cell-cycle, we can judge by the following criteria whether a set of parameter has enabled the ODE system to fulfill it. For each node, a score 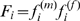 was given to quantify the simulation's resemblance of the target Boolean trajectory, with
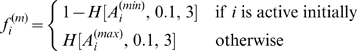
(4)and similarly
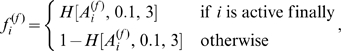
(5)where 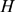 denotes the Hill function defined by equation (2), while the activity of a node refers to that in the Boolean sequence, and 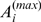, 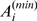 and 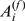 are the maximal, minimal and final value of 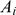 in the (continuous) time course, respectively. We note that the credibility of this score function, which places no weight on the order of extrema but only their amplitudes, is limited to the trajectories produced by networks that *can* fulfill the target sequence in the Boolean approximation.

Then we used the average 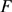 of individual scores to represent the degree of function fulfillment. Calculations showed that 0.4 happened to be a rough cutoff for the top 2% on the tail of the 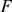 distribution for the two yeast cell-cycle networks, so we defined the fulfillment of function as having a score higher than 0.4, and finally counted out the 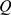 value as the ratio of the parameter sets fulfilling the given function. It should be noted that general results hardly depend on the exact choice of such thresholds or cutoffs, and the above procedure can be readily applied to our construction of random trajectories.

## Supporting Information

Table S1 The cell-cycle control network of the budding yeast
(0.06 MB PDF)

Table S2 The cell-cycle control network of the fission yeast
(0.06 MB PDF)

Table S3 The guard cell abscisic acid signaling network (ABA)
(0.06 MB PDF)

Table S4 The T cell receptor signaling network (TCR)
(0.06 MB PDF)

Table S5 The survival signaling network in T cell large granular lymphocyte leukemia (T-LGL)
(0.06 MB PDF)

Table S6 The network of physical interactions between nuclear proteins in the budding yeast (PI)
(0.06 MB PDF)
