# Supplementary material for: Identification of a Topological Characteristic Responsible for the Biological Robustness of Regulatory Networks
Source: PLoS Comput Biol. 2009 Jul 24;5(7):e1000442. doi: 10.1371/journal.pcbi.1000442 (PMC2704863; doi:10.1371/journal.pcbi.1000442)
Supplement: Table S1 — The cell-cycle control network of the budding yeast (0.06 MB PDF) [file pcbi.1000442.s001.pdf]

Identification of a Topological Characteristic Responsible for the Biological Robustness of Regulatory Networks

Yangle Wu[1,2], Xiaomeng Zhang[1,2], Jianglei Yu[1,2], Qi Ouyang[1,2,3,*]
1 Center for Theoretical Biology, Academy for Advanced Interdisciplinary Studies, Peking University, Beijing, China
2 The State Key Laboratory for Artificial Microstructures and Mesoscopic Physics, School of Physics, Peking University, Beijing, China
3 Department of Physics, Hong Kong Baptist University, Kowloon Tong, Hong Kong
* E-mail: qi@pku.edu.cn

Table S1: The cell-cycle control network of the budding yeast

(Based on Ref. [1])

Cln3	$\xrightarrow{-}$	Cln3	Cln3	$\xrightarrow{+}$	MBF
Cln3	$\xrightarrow{+}$	SBF	MBF	$\xrightarrow{+}$	Clb5,6
SBF	$\xrightarrow{+}$	Cln1,2	Clb5,6	$\xrightarrow{-}$	Cdh1
Clb5,6	$\xrightarrow{-}$	Sic1	Clb5,6	$\xrightarrow{+}$	Clb1,2
Clb5,6	$\xrightarrow{+}$	Mcm1	Cln1,2	$\xrightarrow{-}$	Cln1,2
Cln1,2	$\xrightarrow{-}$	Cdh1	Cln1,2	$\xrightarrow{-}$	Sic1
Cdh1	$\xrightarrow{-}$	Clb1,2	Sic1	$\xrightarrow{-}$	Clb5,6
Sic1	$\xrightarrow{-}$	Clb1,2	Clb1,2	$\xrightarrow{-}$	MBF
Clb1,2	$\xrightarrow{-}$	SBF	Clb1,2	$\xrightarrow{-}$	Cdh1
Clb1,2	$\xrightarrow{-}$	Sic1	Clb1,2	$\xrightarrow{-}$	Swi5
Clb1,2	$\xrightarrow{+}$	Cdc20	Clb1,2	$\xrightarrow{+}$	Mcm1
Swi5	$\xrightarrow{+}$	Sic1	Swi5	$\xrightarrow{-}$	Swi5
Cdc20	$\xrightarrow{-}$	Clb5,6	Cdc20	$\xrightarrow{+}$	Cdh1
Cdc20	$\xrightarrow{+}$	Sic1	Cdc20	$\xrightarrow{-}$	Clb1,2
Cdc20	$\xrightarrow{+}$	Swi5	Cdc20	$\xrightarrow{-}$	Cdc20
Mcm1	$\xrightarrow{+}$	Clb1,2	Mcm1	$\xrightarrow{+}$	Swi5
Mcm1	$\xrightarrow{+}$	Cdc20	Mcm1	$\xrightarrow{-}$	Mcm1

1. Li F, Long T, Lu Y, Ouyang Q, Tang C (2004) The yeast cell-cycle network is robustly designed. Proc Natl Acad Sci U S A 101:4781–4786.
