# Supplementary material for: Identification of a Topological Characteristic Responsible for the Biological Robustness of Regulatory Networks
Source: PLoS Comput Biol. 2009 Jul 24;5(7):e1000442. doi: 10.1371/journal.pcbi.1000442 (PMC2704863; doi:10.1371/journal.pcbi.1000442)
Supplement: Table S2 — The cell-cycle control network of the fission yeast (0.06 MB PDF) [file pcbi.1000442.s002.pdf]

Identification of a Topological Characteristic Responsible for the Biological Robustness of Regulatory Networks

Yangle Wu[1,2], Xiaomeng Zhang[1,2], Jianglei Yu[1,2], Qi Ouyang[1,2,3,*]
1 Center for Theoretical Biology, Academy for Advanced Interdisciplinary Studies, Peking University, Beijing, China
2 The State Key Laboratory for Artificial Microstructures and Mesoscopic Physics, School of Physics, Peking University, Beijing, China
3 Department of Physics, Hong Kong Baptist University, Kowloon Tong, Hong Kong
* E-mail: qi@pku.edu.cn

Table S2: The cell-cycle control network of the fission yeast

(Based on Ref. [1])

Start	$\xrightarrow{-}$	Start	Start	$\xrightarrow{+}$	SK
SK	$\xrightarrow{-}$	SK	SK	$\xrightarrow{-}$	Ste9
SK	$\xrightarrow{-}$	Rum1	Ste9	$\xrightarrow{-}$	Cdc2,13
Ste9	$\xrightarrow{-}$	Cdc2,13*	Rum1	$\xrightarrow{-}$	Cdc2,13
Rum1	$\xrightarrow{-}$	Cdc2,13*	Cdc2,13	$\xrightarrow{-}$	Ste9
Cdc2,13	$\xrightarrow{-}$	Rum1	Cdc2,13	$\xrightarrow{+}$	Cdc2,13
Cdc2,13	$\xrightarrow{-}$	Wee1,Mik1	Cdc2,13	$\xrightarrow{+}$	Cdc25
Wee1,Mik1	$\xrightarrow{-}$	Cdc2,13*	Cdc25	$\xrightarrow{+}$	Cdc2,13*
Cdc2,13*	$\xrightarrow{-}$	Ste9	Cdc2,13*	$\xrightarrow{-}$	Rum1
Cdc2,13*	$\xrightarrow{-}$	Cdc2,13*	Cdc2,13*	$\xrightarrow{+}$	Slp1
Slp1	$\xrightarrow{-}$	Cdc2,13	Slp1	$\xrightarrow{-}$	Cdc2,13*
Slp1	$\xrightarrow{-}$	Slp1	Slp1	$\xrightarrow{+}$	PP
PP	$\xrightarrow{+}$	Ste9	PP	$\xrightarrow{+}$	Rum1
PP	$\xrightarrow{+}$	Wee1,Mik1	PP	$\xrightarrow{-}$	Cdc25
PP	$\xrightarrow{-}$	PP			

1. Davidich MI, Bornholdt S (2008) Boolean network model predicts cell cycle sequence of fission yeast. PLoS ONE 3:e1672.
