# Supplementary material for: Identification of a Topological Characteristic Responsible for the Biological Robustness of Regulatory Networks
Source: PLoS Comput Biol. 2009 Jul 24;5(7):e1000442. doi: 10.1371/journal.pcbi.1000442 (PMC2704863; doi:10.1371/journal.pcbi.1000442)
Supplement: Table S3 — The guard cell abscisic acid signaling network (ABA) (0.06 MB PDF) [file pcbi.1000442.s003.pdf]

Identification of a Topological Characteristic Responsible for the Biological Robustness of Regulatory Networks

Yangle Wu[1,2], Xiaomeng Zhang[1,2], Jianglei Yu[1,2], Qi Ouyang[1,2,3,*]
1 Center for Theoretical Biology, Academy for Advanced Interdisciplinary Studies, Peking University, Beijing, China
2 The State Key Laboratory for Artificial Microstructures and Mesoscopic Physics, School of Physics, Peking University, Beijing, China
3 Department of Physics, Hong Kong Baptist University, Kowloon Tong, Hong Kong
∗ E-mail: qi@pku.edu.cn

Table S3: The guard cell abscisic acid signaling network (ABA)

(Based on Refs. [1, 2])

ABA	$\xrightarrow{+}$	SphK	ABA	$\xrightarrow{+}$	pHc
ABA	$\xrightarrow{+}$	OST1	ABA	$\xrightarrow{+}$	RCN1
ABA	$\xrightarrow{+}$	PLC	ABA	$\xrightarrow{+}$	InsPK
ABA	$\xrightarrow{-}$	PEPC	ABA	$\xrightarrow{-}$	Malate
ABA	$\xrightarrow{-}$	RAC1	SphK	$\xrightarrow{+}$	S1P
S1P	$\xrightarrow{+}$	GPA1	GPA1	$\xrightarrow{+}$	PLD
PLD	$\xrightarrow{+}$	PA	PA	$\xrightarrow{+}$	ROP2
PA	$\xrightarrow{-}$	ABI1	pHc	$\xrightarrow{+}$	Atrboh
pHc	$\xrightarrow{+}$	ABI1	pHc	$\xrightarrow{-}$	H+ATPase
pHc	$\xrightarrow{+}$	AnionEM	pHc	$\xrightarrow{+}$	KOUT
pHc	$\xrightarrow{-}$	KAP	OST1	$\xrightarrow{+}$	Atrboh
ROP2	$\xrightarrow{+}$	Atrboh	Atrboh	$\xrightarrow{+}$	ROS
ABI1	$\xrightarrow{-}$	Atrboh	ABI1	$\xrightarrow{-}$	AnionEM
ABI1	$\xrightarrow{-}$	RAC1	ROS	$\xrightarrow{-}$	ABI1
ROS	$\xrightarrow{-}$	H+ATPase	ROS	$\xrightarrow{+}$	CaIM
ROS	$\xrightarrow{-}$	KOUT	H+ATPase	$\xrightarrow{-}$	Depolar
Ca2+c	$\xrightarrow{-}$	H+ATPase	Ca2+c	$\xrightarrow{+}$	NOS
Ca2+c	$\xrightarrow{+}$	PLC	Ca2+c	$\xrightarrow{+}$	Ca2+ATPase
Ca2+c	$\xrightarrow{+}$	AnionEM	Ca2+c	$\xrightarrow{+}$	KEV
Ca2+c	$\xrightarrow{+}$	Depolar	Ca2+c	$\xrightarrow{-}$	KAP
Ca2+c	$\xrightarrow{+}$	Actin	RCN1	$\xrightarrow{+}$	NIA12
NIA12	$\xrightarrow{+}$	NO	NOS	$\xrightarrow{+}$	NO
NO	$\xrightarrow{+}$	GC	NO	$\xrightarrow{+}$	ADPRc
NO	$\xrightarrow{-}$	KOUT	GC	$\xrightarrow{+}$	cGMP
ADPRc	$\xrightarrow{+}$	cADPR	cADPR	$\xrightarrow{+}$	CIS
cGMP	$\xrightarrow{+}$	CIS	PLC	$\xrightarrow{+}$	InsP3
InsP3	$\xrightarrow{+}$	CIS	InsPK	$\xrightarrow{+}$	InsP6
InsP6	$\xrightarrow{+}$	CIS	CIS	$\xrightarrow{+}$	Ca2+c
Ca2+ATPase	$\xrightarrow{-}$	Ca2+c	CaIM	$\xrightarrow{+}$	Ca2+c
AnionEM	$\xrightarrow{+}$	Depolar	AnionEM	$\xrightarrow{-}$	Malate
AnionEM	$\xrightarrow{+}$	Closure	KEV	$\xrightarrow{+}$	Depolar
Depolar	$\xrightarrow{-}$	CaIM	Depolar	$\xrightarrow{+}$	KOUT
Depolar	$\xrightarrow{+}$	KAP	KOUT	$\xrightarrow{-}$	Depolar
KOUT	$\xrightarrow{+}$	Closure	KAP	$\xrightarrow{+}$	Closure
PEPC	$\xrightarrow{+}$	Malate	Malate	$\xrightarrow{-}$	Closure

(Continued on the next page.)

(Table S3 continued)

RAC1	$\xrightarrow{-}$	Actin	Actin	$\xrightarrow{+}$	Closure

1. Li S, Assmann SM, Albert R (2006) Predicting essential components of signal transduction networks: A dynamic model of guard cell abscisic acid signaling. PLoS Biol 4:e312.
2. Albert I, Thakar J, Li S, Zhang R, Albert R (2008) Boolean network simulations for life scientists. Source Code Biol Med 3:16.
