# Supplementary material for: Identification of a Topological Characteristic Responsible for the Biological Robustness of Regulatory Networks
Source: PLoS Comput Biol. 2009 Jul 24;5(7):e1000442. doi: 10.1371/journal.pcbi.1000442 (PMC2704863; doi:10.1371/journal.pcbi.1000442)
Supplement: Table S4 — The T cell receptor signaling network (TCR) (0.06 MB PDF) [file pcbi.1000442.s004.pdf]

# Identification of a Topological Characteristic Responsible for the Biological Robustness of Regulatory Networks

Yangle Wu[1,2], Xiaomeng Zhang[1,2], Jianglei Yu[1,2], Qi Ouyang[1,2,3,*]
**1** Center for Theoretical Biology, Academy for Advanced Interdisciplinary Studies, Peking University, Beijing, China
**2** The State Key Laboratory for Artificial Microstructures and Mesoscopic Physics, School of Physics, Peking University, Beijing, China
**3** Department of Physics, Hong Kong Baptist University, Kowloon Tong, Hong Kong
* E-mail: qi@pku.edu.cn

Table S4: The T cell receptor signaling network (TCR)

(Based on Ref. [1])

| | | | | | |
|---|---|---|---|---|---|
| bad | $\xrightarrow{-}$ | bclxl | cabin1 | $\xrightarrow{-}$ | calcin |
| calcin | $\xrightarrow{+}$ | nfat | calpr1 | $\xrightarrow{-}$ | calcin |
| akap79 | $\xrightarrow{-}$ | calcin | cam | $\xrightarrow{+}$ | calcin |
| cam | $\xrightarrow{+}$ | camk4 | cam | $\xrightarrow{+}$ | camk2 |
| camk4 | $\xrightarrow{-}$ | cabin1 | card11a | $\xrightarrow{+}$ | ikkg |
| ikkg | $\xrightarrow{+}$ | ikkab | pkcth | $\xrightarrow{+}$ | ikkg |
| ccblp1 | $\xrightarrow{-}$ | tcrb | ccblp1 | $\xrightarrow{-}$ | zap70 |
| tcrb | $\xrightarrow{+}$ | tcrp | tcrb | $\xrightarrow{+}$ | dgk |
| tcrb | $\xrightarrow{-}$ | pag | tcrb | $\xrightarrow{+}$ | fyn |
| tcrb | $\xrightarrow{+}$ | lckp2 | tcrlig | $\xrightarrow{+}$ | tcrb |
| zap70 | $\xrightarrow{+}$ | ccblp1 | zap70 | $\xrightarrow{+}$ | plcga |
| zap70 | $\xrightarrow{+}$ | gab2 | zap70 | $\xrightarrow{+}$ | slp76 |
| zap70 | $\xrightarrow{+}$ | p38 | zap70 | $\xrightarrow{+}$ | vav1 |
| zap70 | $\xrightarrow{+}$ | lat | zap70 | $\xrightarrow{+}$ | itk |
| zap70 | $\xrightarrow{+}$ | sh3bp2 | tcrp | $\xrightarrow{+}$ | zap70 |
| abl | $\xrightarrow{+}$ | zap70 | cd28 | $\xrightarrow{-}$ | cblb |
| cd28 | $\xrightarrow{+}$ | x | cblb | $\xrightarrow{-}$ | pi3k |
| dgk | $\xrightarrow{-}$ | dag | dag | $\xrightarrow{+}$ | pkcth |
| dag | $\xrightarrow{+}$ | rasgrp | plcga | $\xrightarrow{+}$ | dag |
| plcga | $\xrightarrow{+}$ | ip3 | erk | $\xrightarrow{-}$ | shp1 |
| erk | $\xrightarrow{+}$ | fos | erk | $\xrightarrow{+}$ | rsk |
| shp1 | $\xrightarrow{-}$ | lckp1 | lckp1 | $\xrightarrow{+}$ | tcrp |
| lckp1 | $\xrightarrow{+}$ | abl | lckp1 | $\xrightarrow{+}$ | shp1 |
| lckp1 | $\xrightarrow{+}$ | fyn | lckp1 | $\xrightarrow{+}$ | rlk |
| gab2 | $\xrightarrow{-}$ | slp76 | gab2 | $\xrightarrow{+}$ | shp2 |
| slp76 | $\xrightarrow{+}$ | plcga | slp76 | $\xrightarrow{+}$ | itk |
| gads | $\xrightarrow{+}$ | gab2 | gads | $\xrightarrow{+}$ | slp76 |
| gsk3 | $\xrightarrow{-}$ | bcat | gsk3 | $\xrightarrow{-}$ | cyc1 |
| ikb | $\xrightarrow{-}$ | nfkb | ikkab | $\xrightarrow{-}$ | ikb |
| camk2 | $\xrightarrow{+}$ | ikkab | pkb | $\xrightarrow{-}$ | bad |
| pkb | $\xrightarrow{-}$ | gsk3 | pkb | $\xrightarrow{-}$ | fkhr |
| pkb | $\xrightarrow{-}$ | p21c | pkb | $\xrightarrow{-}$ | p27k |
| gadd45 | $\xrightarrow{-}$ | p38 | cd45 | $\xrightarrow{+}$ | lckp1 |
| cd45 | $\xrightarrow{+}$ | fyn | cd4 | $\xrightarrow{+}$ | lckp1 |
| csk | $\xrightarrow{-}$ | lckp1 | lckr | $\xrightarrow{+}$ | lckp1 |

(Continued on the next page.)

(Table S4 continued)

| | | | | | |
|---|---|---|---|---|---|
| lckr | $\xrightarrow{+}$ | fyn | lckr | $\xrightarrow{+}$ | lckp2 |
| pag | $\xrightarrow{+}$ | csk | ca | $\xrightarrow{+}$ | cam |
| ccblr | $\xrightarrow{+}$ | ccblp1 | ccblr | $\xrightarrow{+}$ | ccblp2 |
| ccblp2 | $\xrightarrow{-}$ | plcga | fyn | $\xrightarrow{+}$ | tcrp |
| fyn | $\xrightarrow{+}$ | abl | fyn | $\xrightarrow{+}$ | pag |
| fyn | $\xrightarrow{+}$ | ccblp2 | x | $\xrightarrow{+}$ | vav1 |
| x | $\xrightarrow{+}$ | pi3k | vav1 | $\xrightarrow{+}$ | pkcth |
| vav1 | $\xrightarrow{+}$ | plcga | vav1 | $\xrightarrow{+}$ | rac1p1 |
| cdc42 | $\xrightarrow{+}$ | mekk1 | cdc42 | $\xrightarrow{+}$ | sre |
| mekk1 | $\xrightarrow{+}$ | p38 | mekk1 | $\xrightarrow{+}$ | jnk |
| mekk1 | $\xrightarrow{+}$ | mkk4 | creb | $\xrightarrow{+}$ | cre |
| rasgrp | $\xrightarrow{+}$ | ras | pdk1 | $\xrightarrow{+}$ | pkcth |
| pdk1 | $\xrightarrow{+}$ | pkb | pdk1 | $\xrightarrow{+}$ | p70s |
| fos | $\xrightarrow{+}$ | ap1 | rsk | $\xrightarrow{+}$ | creb |
| jun | $\xrightarrow{+}$ | ap1 | lat | $\xrightarrow{+}$ | gab2 |
| lat | $\xrightarrow{+}$ | gads | lat | $\xrightarrow{+}$ | grb2 |
| lat | $\xrightarrow{+}$ | hpk1 | lat | $\xrightarrow{+}$ | plcgb |
| lat | $\xrightarrow{+}$ | sh3bp2 | grb2 | $\xrightarrow{+}$ | gab2 |
| grb2 | $\xrightarrow{+}$ | sos | hpk1 | $\xrightarrow{+}$ | mekk1 |
| hpk1 | $\xrightarrow{+}$ | mlk3 | mlk3 | $\xrightarrow{+}$ | mkk4 |
| ip3 | $\xrightarrow{+}$ | ca | jnk | $\xrightarrow{+}$ | jun |
| plcgb | $\xrightarrow{+}$ | plcga | rlk | $\xrightarrow{+}$ | plcga |
| lckp2 | $\xrightarrow{+}$ | pi3k | pi3k | $\xrightarrow{+}$ | pip3 |
| malt1 | $\xrightarrow{+}$ | card11a | card11 | $\xrightarrow{+}$ | card11a |
| bcl10 | $\xrightarrow{+}$ | card11a | mek | $\xrightarrow{+}$ | erk |
| mkk4 | $\xrightarrow{+}$ | jnk | pip3 | $\xrightarrow{+}$ | pdk1 |
| pip3 | $\xrightarrow{+}$ | itk | ship1 | $\xrightarrow{-}$ | pip3 |
| pten | $\xrightarrow{-}$ | pip3 | itk | $\xrightarrow{+}$ | plcga |
| rac1p1 | $\xrightarrow{+}$ | mlk3 | rac1p2 | $\xrightarrow{+}$ | mekk1 |
| rac1p2 | $\xrightarrow{+}$ | sre | rac1r | $\xrightarrow{+}$ | rac1p1 |
| rac1r | $\xrightarrow{+}$ | rac1p2 | vav3 | $\xrightarrow{+}$ | rac1p2 |
| raf | $\xrightarrow{+}$ | mek | ras | $\xrightarrow{+}$ | raf |
| sh3bp2 | $\xrightarrow{+}$ | vav1 | sh3bp2 | $\xrightarrow{+}$ | vav3 |
| sos | $\xrightarrow{+}$ | ras | gap | $\xrightarrow{-}$ | ras |

1. Saez-Rodriguez J, Simeoni L, Lindquist JA, Hemenway R, Bommhardt U, et al. (2007) A logical model provides insights into t cell receptor signaling. PLoS Comput Biol 3:e163.
