# Supplementary material for: Identification of a Topological Characteristic Responsible for the Biological Robustness of Regulatory Networks
Source: PLoS Comput Biol. 2009 Jul 24;5(7):e1000442. doi: 10.1371/journal.pcbi.1000442 (PMC2704863; doi:10.1371/journal.pcbi.1000442)
Supplement: Table S5 — The survival signaling network in T cell large granular lymphocyte leukemia (T-LGL) (0.06 MB PDF) [file pcbi.1000442.s005.pdf]

Identification of a Topological Characteristic Responsible for the Biological Robustness of Regulatory Networks

Yangle Wu[1,2], Xiaomeng Zhang[1,2], Jianglei Yu[1,2], Qi Ouyang[1,2,3,*]
1 Center for Theoretical Biology, Academy for Advanced Interdisciplinary Studies, Peking University, Beijing, China
2 The State Key Laboratory for Artificial Microstructures and Mesoscopic Physics, School of Physics, Peking University, Beijing, China
3 Department of Physics, Hong Kong Baptist University, Kowloon Tong, Hong Kong
* E-mail: qi@pku.edu.cn

Table S5: The survival signaling network in T cell large granular lymphocyte leukemia (T-LGL)

(Based on Refs. [1, 2])

BclxL	$\xrightarrow{-}$	BID	BID	$\xrightarrow{-}$	BclxL
BID	$\xrightarrow{+}$	Ceramide	Ceramide	$\xrightarrow{-}$	Flip
Ceramide	$\xrightarrow{+}$	Fas	Caspase	$\xrightarrow{+}$	BID
Caspase	$\xrightarrow{+}$	Apoptosis	CD45	$\xrightarrow{+}$	LCK
CD45	$\xrightarrow{-}$	JAK	LCK	$\xrightarrow{+}$	GRB2
JAK	$\xrightarrow{+}$	JAK	JAK	$\xrightarrow{+}$	IFNT
JAK	$\xrightarrow{+}$	SOCS	JAK	$\xrightarrow{+}$	STAT3
Flip	$\xrightarrow{-}$	Caspase	Flip	$\xrightarrow{+}$	NFKB
Fas	$\xrightarrow{+}$	Caspase	Fas	$\xrightarrow{-}$	Mcl1
C-Myc	$\xrightarrow{+}$	Proliferation	C-Myc	$\xrightarrow{+}$	IL2
C-Myc	$\xrightarrow{+}$	FasL	IL2	$\xrightarrow{-}$	BclxL
IL2	$\xrightarrow{-}$	Flip	IL2	$\xrightarrow{+}$	IL2RL
IL2	$\xrightarrow{-}$	Mcl1	IL2	$\xrightarrow{-}$	GAP
IL2	$\xrightarrow{+}$	IFN	IL2	$\xrightarrow{-}$	SOCS
IL2	$\xrightarrow{+}$	IL2R	FasL	$\xrightarrow{+}$	Ceramide
FasL	$\xrightarrow{+}$	Fas	CREB	$\xrightarrow{+}$	Flip
CREB	$\xrightarrow{+}$	GZMB	GZMB	$\xrightarrow{+}$	BID
GZMB	$\xrightarrow{+}$	GZMB	GZMB	$\xrightarrow{-}$	TGF
CSK	$\xrightarrow{-}$	LCK	CTLA4	$\xrightarrow{-}$	TCR
TCR	$\xrightarrow{+}$	LCK	TCR	$\xrightarrow{+}$	CSK
TCR	$\xrightarrow{+}$	CTLA4	Erk	$\xrightarrow{+}$	FasL
Erk	$\xrightarrow{+}$	CREB	Erk	$\xrightarrow{+}$	IL2RL
Erk	$\xrightarrow{+}$	TGF	Erk	$\xrightarrow{+}$	Mcl1
IL2RL	$\xrightarrow{+}$	LCK	IL2RL	$\xrightarrow{+}$	JAK
IL2RL	$\xrightarrow{+}$	Fyn	TGF	$\xrightarrow{+}$	SMAD
Mcl1	$\xrightarrow{-}$	BID	FasT	$\xrightarrow{+}$	Fas
FasT	$\xrightarrow{+}$	FasT	NFKB	$\xrightarrow{+}$	BclxL
NFKB	$\xrightarrow{+}$	Flip	NFKB	$\xrightarrow{+}$	IL2
NFKB	$\xrightarrow{+}$	FasL	NFKB	$\xrightarrow{+}$	Mcl1
NFKB	$\xrightarrow{+}$	FasT	NFKB	$\xrightarrow{+}$	IAP
NFKB	$\xrightarrow{+}$	TNF	NFKB	$\xrightarrow{+}$	RANTES
Fyn	$\xrightarrow{+}$	Migration	GAP	$\xrightarrow{-}$	RAS
RAS	$\xrightarrow{-}$	BclxL	RAS	$\xrightarrow{+}$	GAP
RAS	$\xrightarrow{+}$	MEK	RAS	$\xrightarrow{+}$	PI3K
GRB2	$\xrightarrow{+}$	RAS	GRB2	$\xrightarrow{+}$	PLC

(Continued on the next page.)

(Table S5 continued)

PLC	$\xrightarrow{+}$	RAS	PLC	$\xrightarrow{+}$	NFAT
IAP	$\xrightarrow{-}$	Caspase	IAP	$\xrightarrow{+}$	NFKB
IAP	$\xrightarrow{-}$	TRADD	TRADD	$\xrightarrow{+}$	NFKB
IFN	$\xrightarrow{+}$	Flip	IFN	$\xrightarrow{+}$	CREB
IFN	$\xrightarrow{-}$	Erk	IFNT	$\xrightarrow{-}$	BclxL
IFNT	$\xrightarrow{+}$	IFN	IFNT	$\xrightarrow{+}$	TBET
TBET	$\xrightarrow{-}$	IL2	TBET	$\xrightarrow{+}$	GZMB
IL15	$\xrightarrow{-}$	GAP	IL15	$\xrightarrow{-}$	SOCS
SOCS	$\xrightarrow{-}$	JAK	IL2R	$\xrightarrow{-}$	IL2R
STAT3	$\xrightarrow{+}$	BclxL	STAT3	$\xrightarrow{+}$	C-Myc
STAT3	$\xrightarrow{+}$	Mcl1	STAT3	$\xrightarrow{+}$	IL2R
STAT3	$\xrightarrow{-}$	PIAS	MEK	$\xrightarrow{+}$	Erk
NFAT	$\xrightarrow{+}$	IL2	NFAT	$\xrightarrow{+}$	FasL
TNF	$\xrightarrow{-}$	TGF	TNF	$\xrightarrow{+}$	TRADD
TNF	$\xrightarrow{+}$	TPL2	PI3K	$\xrightarrow{+}$	NFKB
PI3K	$\xrightarrow{+}$	TNF	PIAS	$\xrightarrow{-}$	NFKB
sFas	$\xrightarrow{-}$	Fas	sFas	$\xrightarrow{+}$	sFas
SMAD	$\xrightarrow{-}$	C-Myc	SMAD	$\xrightarrow{-}$	IL2
SMAD	$\xrightarrow{+}$	TGF	SMAD	$\xrightarrow{-}$	IFN
SMAD	$\xrightarrow{+}$	SMAD7	SMAD7	$\xrightarrow{-}$	SMAD
Stimuli	$\xrightarrow{+}$	TCR	Stimuli	$\xrightarrow{+}$	IFN
Stimuli	$\xrightarrow{+}$	Stimuli	TPL2	$\xrightarrow{+}$	NFKB
TPL2	$\xrightarrow{+}$	MEK			

1. Zhang R, Shah MV, Yang J, Nyland SB, Liu X, et al. (2008) Network model of survival signaling in large granular lymphocyte leukemia. Proc Natl Acad Sci U S A 105:16308–16313.
2. Albert I, Thakar J, Li S, Zhang R, Albert R (2008) Boolean network simulations for life scientists. Source Code Biol Med 3:16.
