# Supplementary material for: Identification of a Topological Characteristic Responsible for the Biological Robustness of Regulatory Networks
Source: PLoS Comput Biol. 2009 Jul 24;5(7):e1000442. doi: 10.1371/journal.pcbi.1000442 (PMC2704863; doi:10.1371/journal.pcbi.1000442)
Supplement: Table S6 — The network of physical interactions between nuclear proteins in the budding yeast (PI) (0.06 MB PDF) [file pcbi.1000442.s006.pdf]

Identification of a Topological Characteristic Responsible for the Biological Robustness of Regulatory Networks

Yangle Wu[1,2], Xiaomeng Zhang[1,2], Jianglei Yu[1,2], Qi Ouyang[1,2,3,*]
1 Center for Theoretical Biology, Academy for Advanced Interdisciplinary Studies, Peking University, Beijing, China
2 The State Key Laboratory for Artificial Microstructures and Mesoscopic Physics, School of Physics, Peking University, Beijing, China
3 Department of Physics, Hong Kong Baptist University, Kowloon Tong, Hong Kong
* E-mail: qi@pku.edu.cn

Table S6: The network of physical interactions between nuclear proteins in the budding yeast (PI)

(Based on Refs. [1, 2])

CAT5 $\xrightarrow{+}$ CAT5	CAT5 $\xrightarrow{+}$ CAT8
CAT8 $\xrightarrow{+}$ CAT5	MIG2 $\xrightarrow{-}$ CAT8
MIG2 $\xrightarrow{-}$ HXT13	HXT13 $\xrightarrow{-}$ HXT13
SNF1 $\xrightarrow{+}$ CAT5	SNF1 $\xrightarrow{-}$ MIG1
MIG1 $\xrightarrow{-}$ CAT8	MIG1 $\xrightarrow{-}$ HXT13
MIG1 $\xrightarrow{-}$ HAP2	MIG1 $\xrightarrow{-}$ HAP4
MIG1 $\xrightarrow{-}$ GAL4	MIG1 $\xrightarrow{-}$ HXK1
HAP2 $\xrightarrow{+}$ HEM1	HAP4 $\xrightarrow{+}$ HEM1
GAL4 $\xrightarrow{+}$ GAL80	HXK1 $\xrightarrow{-}$ HXK2
HXK2 $\xrightarrow{-}$ HXK1	HXK2 $\xrightarrow{+}$ HXK2
GAL80 $\xrightarrow{-}$ GAL80	MSN4 $\xrightarrow{+}$ HXK1
MSN4 $\xrightarrow{+}$ RAS2	HOG1 $\xrightarrow{+}$ HXK1
HOG1 $\xrightarrow{+}$ RAS2	MSN2 $\xrightarrow{+}$ HXK1
MSN2 $\xrightarrow{+}$ RAS2	RAS2 $\xrightarrow{+}$ STE7
HEM1 $\xrightarrow{+}$ ROX1	SWI1 $\xrightarrow{+}$ HAP4
SWI1 $\xrightarrow{+}$ MCM1	SNF2 $\xrightarrow{+}$ HAP4
SNF2 $\xrightarrow{+}$ MCM1	MCM1 $\xrightarrow{+}$ SWI5
HAP3 $\xrightarrow{+}$ HEM1	HAP5 $\xrightarrow{+}$ HEM1
ROX1 $\xrightarrow{-}$ ROX1	HAP1 $\xrightarrow{+}$ ROX1
TUP1 $\xrightarrow{-}$ ROX1	TUP1 $\xrightarrow{-}$ RME1
TUP1 $\xrightarrow{-}$ IME1	SWI5 $\xrightarrow{+}$ RME1
NDD1 $\xrightarrow{+}$ SWI5	REB1 $\xrightarrow{+}$ SWI5
REB1 $\xrightarrow{+}$ SIN3	SIN3 $\xrightarrow{+}$ SWI1
SIN3 $\xrightarrow{+}$ RME1	RME1 $\xrightarrow{-}$ IME1
RME1 $\xrightarrow{+}$ CLN2	ACE2 $\xrightarrow{+}$ RME1
IME1 $\xrightarrow{-}$ IME1	IME1 $\xrightarrow{+}$ IME2
IME1 $\xrightarrow{+}$ RIM11	IME1 $\xrightarrow{+}$ RIM4
UME6 $\xrightarrow{-}$ RME1	UME6 $\xrightarrow{-}$ IME1
UME6 $\xrightarrow{-}$ IME2	UME6 $\xrightarrow{-}$ RIM4
UME6 $\xrightarrow{+}$ INO2	RIM8 $\xrightarrow{+}$ IME1
RIM13 $\xrightarrow{+}$ IME1	RIM9 $\xrightarrow{+}$ IME1
RPN4 $\xrightarrow{+}$ RPN1	RPN1 $\xrightarrow{+}$ IME1
RIM101 $\xrightarrow{+}$ IME1	IME4 $\xrightarrow{+}$ IME1
MCK1 $\xrightarrow{+}$ IME1	RIM15 $\xrightarrow{+}$ IME1
RIM15 $\xrightarrow{+}$ IME2	IME2 $\xrightarrow{-}$ IME1

(Continued on the next page.)

(Table S6 continued)

RIM11 $\xrightarrow{+}$ IME1	RIM11 $\xrightarrow{+}$ IME2
RIM11 $\xrightarrow{+}$ RIM4	RIM4 $\xrightarrow{+}$ IME2
SDS3 $\xrightarrow{-}$ IME2	RPD3 $\xrightarrow{-}$ IME2
CBK1 $\xrightarrow{-}$ IME2	CLN2 $\xrightarrow{+}$ CLN2
INO2 $\xrightarrow{+}$ INO2	INO2 $\xrightarrow{+}$ CDS1
INO2 $\xrightarrow{+}$ PGS1	CDS1 $\xrightarrow{-}$ PGS1
OPI1 $\xrightarrow{-}$ INO2	OPI1 $\xrightarrow{-}$ PGS1
PGS1 $\xrightarrow{+}$ PGS1	RHO $\xrightarrow{+}$ PGS1
CRD1 $\xrightarrow{+}$ PGS1	INO4 $\xrightarrow{+}$ INO2
INO4 $\xrightarrow{+}$ CDS1	INO4 $\xrightarrow{+}$ PGS1
INO4 $\xrightarrow{+}$ INO4	HYM1 $\xrightarrow{-}$ IME2
STE12 $\xrightarrow{+}$ HYM1	STE12 $\xrightarrow{+}$ STE12
STE12 $\xrightarrow{+}$ FAR1	STE12 $\xrightarrow{+}$ CLN1
BCK2 $\xrightarrow{+}$ CLN2	FAR1 $\xrightarrow{-}$ CLN2
CLN1 $\xrightarrow{+}$ CLN2	SIT4 $\xrightarrow{+}$ CLN2
SIT4 $\xrightarrow{+}$ CLN1	SWI6 $\xrightarrow{+}$ CLN2
SWI6 $\xrightarrow{+}$ CLN1	SWI6 $\xrightarrow{+}$ SWI4
SWI4 $\xrightarrow{+}$ CLN2	SWI4 $\xrightarrow{+}$ CLN1
SPT16 $\xrightarrow{+}$ CLN2	SPT16 $\xrightarrow{+}$ CLN1
SPT16 $\xrightarrow{+}$ SWI6	SPT16 $\xrightarrow{+}$ SWI4
SPT16 $\xrightarrow{+}$ SPT16	SPT16 $\xrightarrow{+}$ CLN3
SPT16 $\xrightarrow{+}$ HTA1	SPT16 $\xrightarrow{+}$ HTB1
CLN3 $\xrightarrow{+}$ CLN2	CLN3 $\xrightarrow{+}$ CLN1
CLN3 $\xrightarrow{+}$ SWI4	STE7 $\xrightarrow{+}$ CLN1
TEC1 $\xrightarrow{+}$ CLN1	XBP $\xrightarrow{-}$ CLN1
XBP $\xrightarrow{-}$ CLN3	PDR1 $\xrightarrow{+}$ XBP
PDR1 $\xrightarrow{+}$ PDR3	PDR1 $\xrightarrow{+}$ YRR1
PDR3 $\xrightarrow{+}$ PDR3	YRR1 $\xrightarrow{+}$ YRR1
HTA1 $\xrightarrow{+}$ HTB1	HTB1 $\xrightarrow{+}$ HTB1
SPT5 $\xrightarrow{+}$ HTA1	SPT5 $\xrightarrow{+}$ HTB1
SPT10 $\xrightarrow{+}$ HTA1	SPT21 $\xrightarrow{+}$ HTA1
SPT4 $\xrightarrow{+}$ HTA1	SPT4 $\xrightarrow{+}$ HTB1
SPT6 $\xrightarrow{+}$ HTA1	SPT6 $\xrightarrow{+}$ HTB1
HTA2 $\xrightarrow{+}$ HTB1	

1. Li FT, Jia X (2006) Dynamical analysis of protein regulatory network in budding yeast nucleus. Chin Phys Lett 23:2307.
2. Maslov S, Sneppen K (2002) Specificity and stability in topology of protein networks. Science 296:910.
